# Nature's laboratory: plant metabolic engineering methods using phenylpropanoids as a case study

**DOI:** 10.1186/s13068-025-02684-9

**Published:** 2025-07-24

**Authors:** Caroline Van Beirs, Ilias El Houari, Bartel Vanholme

**Affiliations:** 1 Department of Plant Biotechnology and Bioinformatics, Ghent University, B–9052 Ghent, Belgium; 2 Department of Plant Systems Biology, VIB, B–9052 Ghent, Belgium; 3 Whitehead Institute for Biomedical Research, Whitehead Institute, 02141 Cambridge, USA; 4 Present Address: Department of Work, Economy, Science, Innovation and Social Economy, Flemish Government, 1000 Brussels, Belgium

**Keywords:** Metabolic engineering, Phenylpropanoids, Specialised metabolites, Plants

## Abstract

Plant specialised metabolism generates a vast array of compounds with significant potential across agriculture, medicine, cosmetics, and the food industry. A key challenge lies in optimising their production in the plant, as these compounds are often present in trace amounts in a complex metabolic cocktail. Given their high economic value, extensive efforts have been made to elucidate their biosynthetic pathways and pinpoint key regulatory and enzymatic targets. This knowledge has been applied for metabolic engineering to enhance the carbon flux towards metabolites of interest, thereby broadening the utility of plants as a source of high-value compounds. This review examines different metabolic engineering strategies employed today using the phenylpropanoid pathway as a case study and highlights the potential of integrating plant and microbial research to drive cross-disciplinary innovation.

## Concerning the potential of plant metabolites

An organism’s metabolome encompasses the complete set of small molecules or metabolites present in or produced by that organism. Metabolites found across a wide range of organisms and essential for fundamental life processes—such as growth, development, and reproduction—are classified as primary metabolites [1]. These include amino acids, nucleotides, sugars and lipids, and can serve as energy sources, signalling molecules, or building blocks for cellular structures [2]. Secondary or specialised metabolites are generally not considered essential for basic survival, but contribute to specific developmental processes or mediate ecological interactions [1, 3, 4]. Compared with primary metabolites, secondary metabolites are generally more structurally complex and are synthesised through specialised biosynthetic pathways, often unique to particular genera or species [5].

The unique chemical diversity and biological activity of specialised plant metabolites give them substantial economic significance across diverse sectors [1, 6, 7]. For instance, they are commonly used in traditional and modern medicine and serve as essential precursors for numerous therapeutic agents [8]. Notable examples include the alkaloids morphine and quinine, which possess potent analgesic and antimalarial properties, respectively, the terpenoid paclitaxel being a cornerstone in anticancer treatments, and salicylic acid, a phenolic compound that laid the groundwork for the development of aspirin [9–12].

The agricultural industry also capitalises on the potential of plant specialised metabolism. Pyrethroids extracted from chrysanthemum flowers and used as biopesticides are an example [13]. Flavonoids, terpenoids, and alkaloids enhance plant resistance to pests, diseases, and environmental stressors, thereby contributing to improved plant resilience [14]. Other metabolites stimulate plant growth by interfering with hormone homeostasis, such as the phenylpropanoid *cis*-cinnamic acid, which stimulates root development by interfering with auxin transport. The resulting expansion in root surface likely enhances nutrient uptake efficiency, increasing biomass [15, 16]. The concentration-dependent activity of these bioactive metabolites, with higher concentrations exhibiting toxicity, also makes them potential herbicides.

The cosmetics and personal care industries extensively utilise plant metabolites for their antioxidant, anti-inflammatory, or UV-protective properties, as these characteristics make them suitable additives in products ranging from skin balms to hair care formulations [6]. Resveratrol, a stilbene found in grapes, is for example widely used in antiaging creams due to its antioxidant properties [17], while terpenoids and essential oils contribute to natural fragrances, making them ideal for use in perfumes, lotions, shampoos, and other personal care products [18]. Plant specialised metabolites also play a crucial role in the food industry (e.g. vanillin, menthol, carotenoids, betanin, and flavonoids), where they are employed as natural flavourings, colourants, and preservatives [19].

Despite the considerable economic potential of plant metabolites, their large-scale production remains a significant challenge, limiting both their accessibility and application. Plant metabolites are often present in trace amounts, making their extraction from plant biomass labour-intensive, often yielding low quantities [20]. For instance, the production of a gram of the cardiac glycoside digoxin, used for the treatment of various heart conditions, requires 4 kg of freeze-dried *Digitalis* leaves [21], and a similar amount of dried *Papaver* capsules is needed to extract a single gram of the analgesic codeine [22]. While these plants can be cultivated, the high demand introduces significant challenges in producing metabolite quantities that meet the needs. In the case of codeine, the annual global consumption is estimated at approximately 250 tons, necessitating an extensive agricultural and processing infrastructure to meet this requirement [23].

In addition to their low abundance, the inherent chemical complexity of plant extracts presents a significant challenge in the purification of target compounds from plant material [24, 25]. Furthermore, the concentration of the desired compound, along with the abundance of other metabolites contributing to the plant’s intricate metabolic profile, can fluctuate substantially. This variability stems from a diverse array of factors, including genetic variation, plant developmental stage, and environmental influences such as seasonal fluctuations, climatic conditions, and geographic location. Together, these challenges hinder the consistent and scalable production of plant metabolites, posing significant obstacles to their commercial utilisation [26–28].

An alternative to biological extraction is chemical synthesis. While structurally simple plant metabolites, such as vanillin, menthol, and coumarin, can be readily synthesised, many economically significant compounds exhibit considerable structural complexity. Features such as chiral centres and multi-ring systems often make chemical synthesis challenging [26, 28]. For these compounds, bioengineering approaches utilising microorganisms such as *Escherichia coli* or *Saccharomyces cerevisiae* have proven to be a viable alternative. In these systems, relevant biosynthetic genes are overexpressed in the host organism, enabling the production of target metabolites. This field, known as white biotechnology, has yielded numerous successes across a wide range of industries and facilitates the production of a broad variety of economically relevant compounds [29].

Despite these achievements, the use of microorganisms can face limitations in producing plant metabolites that require more complex metabolic pathways involving multiple enzymatic reactions [30]. In addition, differences in post-translational modifications can result in diminished or even absent enzyme activity and essential precursors or cofactors may be either absent or present in limited quantities in microbial systems [31]. Moreover, the relatively simple cellular architecture of prokaryotes restricts their capacity to leverage cellular or subcellular compartmentalisation—a mechanism commonly utilised by plants to alleviate metabolite toxicity [32].

Given these considerations, plants often serve as the only potential source for the metabolites of interest. To address challenges such as low compound yields, which can significantly constrain economic feasibility, metabolic engineering has emerged as a powerful tool to improve both the efficiency and scalability of plant-based metabolite production. By leveraging advances in genomics, biotechnology, and synthetic biology, this strategy not only enables the optimisation of native biosynthetic pathways, but also allows the transfer of metabolic routes to potentially more suitable plant species that do not naturally produce the target compounds. The ultimate goal is to unlock the potential of plants to produce valuable and often structurally complex metabolites at high yields without the need for costly equipment or external carbon and energy sources beyond carbon dioxide and sunlight [25]. Supporting this vision, techno-economic analyses have demonstrated that in planta production can surpass microbial synthesis in terms of cost-efficiency and scalability, especially for high-value compounds [33].

## Plant specialised metabolism: a focus on phenylpropanoids

Specialised metabolites are derived from primary metabolites, and although there is a vast diversity of specialised metabolites in plants, only a few key metabolic pathways branch off from primary metabolism. These pathways produce common metabolic intermediates, which are subsequently modified to generate a diverse array of chemically distinct compounds, encompassing various classes such as terpenoids, phenylpropanoids, polyketides, and alkaloids [1]. Here, we will focus on phenylpropanoids to illustrate the different engineering strategies that are employed to optimise the production of specialised metabolites in plants.

Phenylpropanoids and phenylpropanoid-derived compounds constitute a complex and diverse class of specialised plant metabolites, characterised by a core molecular structure composed of a three-carbon side chain attached to a phenyl group [34]. These compounds play pivotal roles in various physiological processes essential to plant survival and adaptation, such as providing structural support, mediating defence responses, and facilitating intracellular and intercellular signalling pathways [35]. Their importance extends beyond plant physiology, as phenylpropanoids are utilised in numerous industrial applications, including pharmaceuticals, food additives, and bio-based materials (Table 1).

**Table 1:**
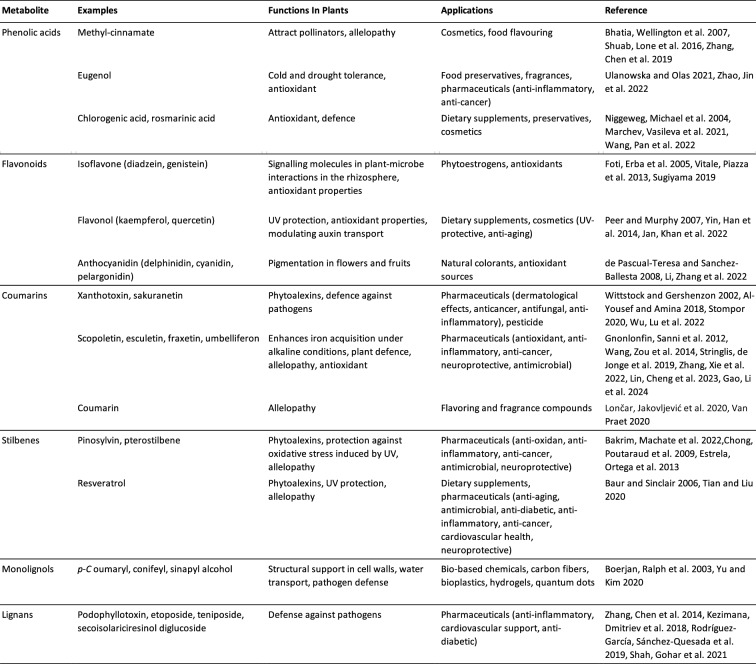
Different phenylpropanoid classes, with representatives for each class, their roles in plant physiology, and their economical relevance

**Fig. 1:**
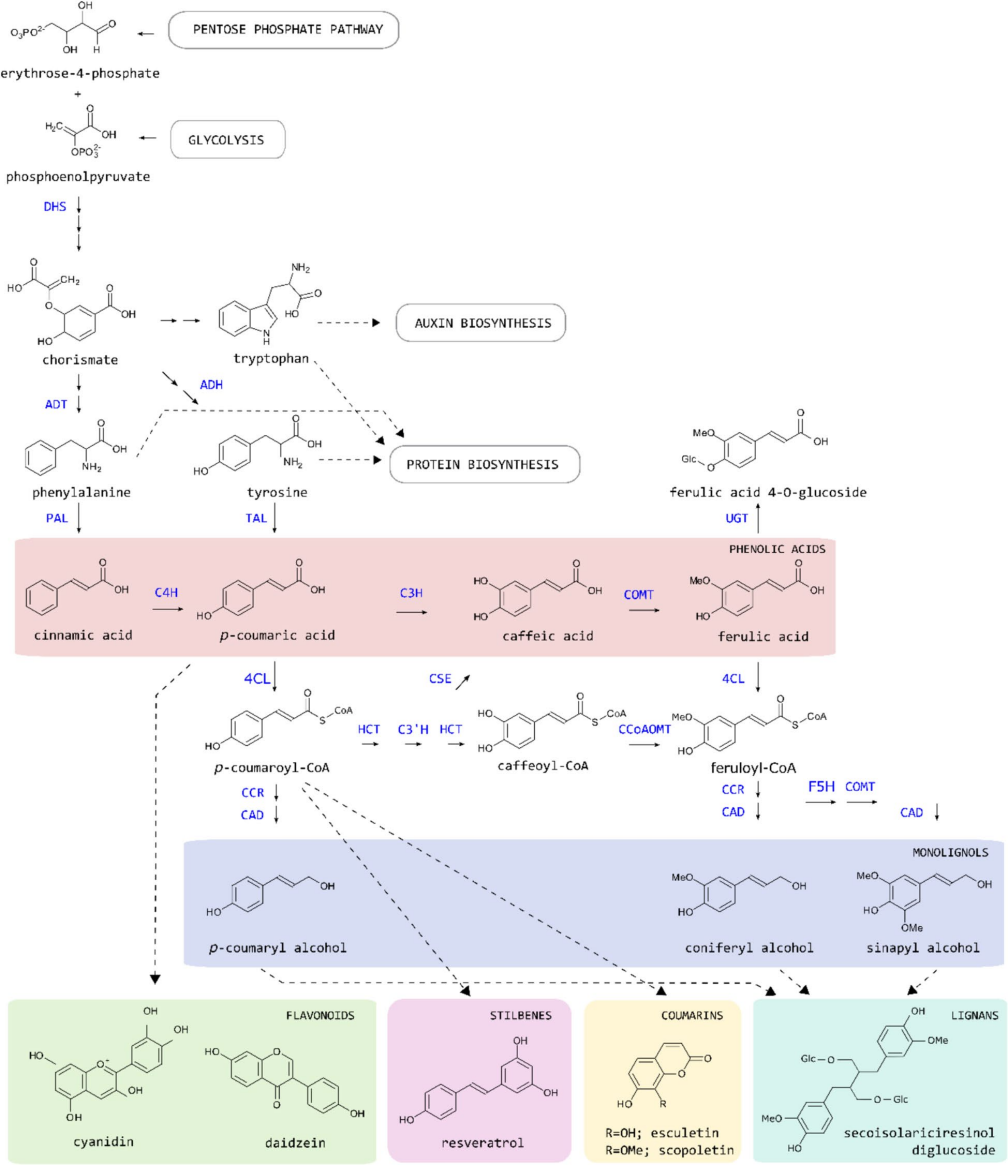
The phenylpropanoid pathway in plants. The phenolic metabolite classes are depicted in coloured frames, and representative metabolites are given for each. Not every phenolic metabolite class shown is present in every plant species. Multiple arrows indicate multiple enzymatic steps. 3-DEOXY-D-ARABINO-HEPTULOSONATE 7-PHOSPHATE SYNTHASE (DHS), 4-COUMARATE: CoA LIGASE (4CL), 4-COUMAROYL SHIKIMATE/QUINATE 3’-HYDROXYLASE (C3’H), AROGENATE DEHYDRATASE (ADT), AROGENATE DEHYDROGENASE (ADH), CAFFEIC ACID *O*-METHYLTRANSFERASE (COMT), CAFFEOYL-CoA *O*-METHYLTRANSFERASE (CCOAOMT), CAFFEOYL SHIKIMATE ESTERASE (CSE), CINNAMATE 4-HYDROXYLASE (C4H), CINNAMOYL-CoA REDUCTASE (CCR), CINNAMYL ALCOHOL DEHYDROGENASE (CAD), FERULATE 5-HYDROXYLASE (F5H), HYDROXYCINNAMOYL-CoA: SHIKIMATE/QUINATE HYDROXYCINNAMOYLTRANSFERASE (HCT), 4-COUMARATE 3-HYDROXYLASE (C3H), PHENYLALANINE AMMONIA-LYASE (PAL), TAL TYROSINE AMMONIA-LYASE (TAL), UDP-GLUCOSYLTRANSFERASE (UGT)

The biosynthesis of phenylpropanoids is initiated by the enzyme PHENYLALANINE AMMONIA LYASE (PAL), which catalyses the deamination of the aromatic amino acid phenylalanine (Fig. 1) [35]. This enzymatic step is significant because it bridges primary metabolic processes with the specialised phenylpropanoid pathway, thus integrating essential biochemical pathways with those involved in specialised metabolism [36]. The product of this enzymatic reaction, cinnamic acid, is converted into *p*-coumaric acid by CINNAMATE-4-HYDROXYLASE (C4H). Next, 4-HYDROXYCINNAMATE-CoA LIGASE (4CL) converts *p*-coumaric acid into *p*-coumaroyl-CoA, which is further converted into *p*-coumaroyl-shikimate by HYDROXYCINNAMOYL-CoA:SHIKIMATE HYDROXYCINNAMOYL TRANSFERASE (HCT) [37]. Afterwards, *p*-COUMAROYL SHIKIMATE/QUINATE 3’HYDROXYLASE (C3’H) hydroxylates caffeoyl shikimate, which is converted into caffeoyl-CoA by HCT again. The formed caffeoyl-CoA is further converted into feruloyl-CoA by CAFFEOYL-CoA *O*-METHYLTRANSFERASE (CCoAOMT). This part of the phenylpropanoid pathway, in which phenylalanine is converted to CoA-esters, is known as the general phenylpropanoid pathway. The second part of the pathway, referred to as the monolignol or lignin-specific pathway, converts the CoA-esters into their corresponding alcohols. As the name suggests, this pathway leads towards the building blocks for lignin, a hydrophobic polymer predominantly deposited in the secondary cell wall of plants [35]. The monolignols *p*-coumaryl alcohol and coniferyl alcohol are produced via the actions of CINNAMOYL-CoA REDUCTASE (CCR) and CINNAMYL ALCOHOL DEHYDROGENASE (CAD), whereas a third major lignin building block, sinapyl alcohol, requires the additional action of FERULATE 5-HYDROXYLASE (F5H) and CAFFEATE *O*-METHYLTRANSFERASE (COMT).

In addition to the conserved central pathway, some plant groups demonstrate flexibility or additional metabolic conversions in their phenylpropanoid pathway. For example, monocots possess a bifunctional PHENYLALANINE TYROSINE AMMONIA LYASE (PTAL), which has both PAL and TYROSINE AMMONIA LYASE (TAL) activity, and which can consequently produce both cinnamic acid and *p*-coumaric acid from phenylalanine and tyrosine, respectively [38]. Consequently, monocots gain an additional entry point into the phenylpropanoid pathway, a feature that is proposed to be linked to the distinct lignin characteristics found in grasses [38]. Further downstream in the pathway, many plants exhibit significant flexibility by converting caffeoyl shikimate into caffeoyl-CoA through a two-step reaction. This process is catalysed by CAFFEOYL SHIKIMATE ESTERASE (CSE) and 4-COUMARATE: CoA LIGASE (4CL), with caffeic acid serving as an intermediate (Fig. 1) [39]. Notably, caffeic acid can also be synthesised from *p*-coumaric acid via a hydroxylation reaction catalysed by 4-COUMARATE-3-HYDROXYLASE (C3H) [40]. Together, these examples underscore the metabolic versatility in lignin biosynthesis, enabling plants to dynamically adjust the flow of intermediates in response to environmental and developmental cues.

When focusing on the side branches of the phenylpropanoid pathway, an even greater degree of flexibility is evident, with certain end products being specific to particular plant species. For instance, capsaicin is exclusively synthesised by plants of the genus *Capsicum* (e.g. chilli peppers), imparting their characteristic spicy flavour, curcumin is responsible for the distinctive yellow colour of turmeric rhizomes (*Curcuma longa*), and rosmarinic acid is predominantly found in plants of the Lamiaceae family contributing to the unique flavour and aroma of the herbs producing it [41–43].

In addition to the species-specific side branches, the phenylpropanoid pathway exhibits developmental and environmental flexibility [36]. Under high UV radiation, for instance, plants upregulate flavonoid biosynthesis to enhance UV shielding and reduce oxidative stress [44]. In response to pathogen attack, the phenylpropanoid pathway shifts towards producing antimicrobial compounds, such as phytoalexins, to bolster the plant's immune defences [45]. Furthermore, environmental stresses, including drought and nutrient deficiency, can stimulate lignin biosynthesis, reinforcing cell walls to protect against cellular damage and water loss [46]. This adaptability highlights the phenylpropanoid pathway’s pivotal role in plant resilience and underscores its evolutionary significance in enabling plants to modulate metabolic responses according to external stressors. The overall pathway flexibility is of significant interest from an engineering perspective, as it provides opportunities to redirect the pathway, channelling carbon skeletons towards the production of target metabolites. Additionally, pathway flexibility facilitates the introduction of non-native enzymes or pathway modules, enabling the synthesis of novel compounds [47].

## Metabolic engineering strategies

**Fig. 2:**
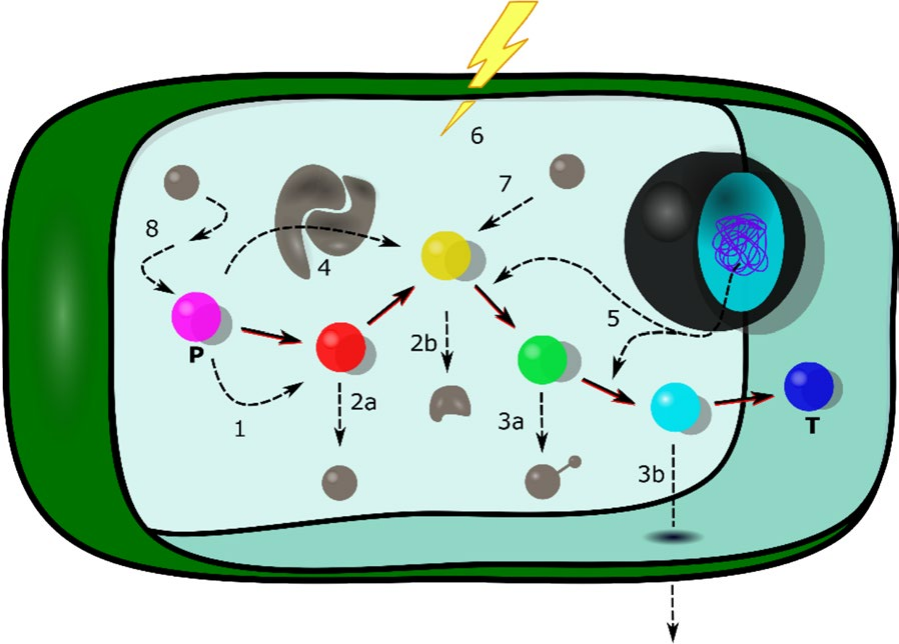
Graphical overview of the described plant metabolic engineering strategies. Hypothetical biosynthetic pathway of interest starting with a precursor (bold P) till the targeted end product (bold T). Coloured spheres represent different metabolites; the large black sphere embodies the nucleus with the DNA (blue); the red/black lines indicate metabolic conversions; the black dotted lines symbolise the effect of the applied metabolic engineering strategy, which is indicated by the different numbers: (1) overcoming rate-limiting steps; (2a) minimising the loss of pathway intermediates through conversion or (2b) degradation; (3a) avoiding toxicity by modification or (3b) transport; (4) facilitating metabolon formation; (5) employing transcription factors to regulate expression; (6) exploiting elicitors to boost production; (7) introducing additional entry points into the pathway; and (8) optimising the supply of precursors

Optimising the carbon flux within a metabolic pathway entails the strategic manipulation of biochemical reactions, enzyme activities, and regulatory networks to maximise the yield of the desired metabolite(s). Figure 2 illustrates a schematic overview of the diverse metabolic engineering strategies employed to engineer metabolic pathway(s).

### Overcoming the rate-limiting step

The overall rate of a biosynthetic pathway is considered to be determined by the slowest or most energetically demanding enzymatic reaction in the pathway, also referred to as the rate-limiting step [48]. By setting the pace for the entire pathway, the rate-limiting step serves as the main control point regulating the carbon flux over the pathway. When excessively slow, it can create a bottleneck and impede the entire biosynthesis process. Conversely, bypassing or accelerating this step can increase the overall rate of the pathway.

In the case of the phenylpropanoid pathway, the rate-limiting step is the first enzymatic conversion of the pathway catalysed by PAL [36]. A first approach to boost the rate-limiting step is to produce more of the enzyme through overexpression of the corresponding gene. The earliest report of this strategy dates back to 1996, and described the expression of the *Phaseolus vulgaris PAL2* in tobacco [49]. This resulted in the accumulation of phenylpropanoids such as *p*-coumaric acid and chlorogenic acid. Interestingly, it did not lead to the accumulation of downstream flavonoids, suggesting the involvement of additional regulatory mechanisms in flavonoid biosynthesis. A more recent study demonstrated that overexpression of several native *PALs* (i.e. *SbPAL1, SbPAL2,* and *SbPAL3*) in hairy roots of the medicinal plant *Scutellaria baicalensis* led to the accumulation of the flavones baicalin, baicalein, and wogonin [50]. Similarly, overexpression of the native *Glycine max PAL2.1* resulted in enhanced PAL activity and increased levels of the flavonoids daidzein and genistein [51].

Besides overexpressing genes underlying the rate-limiting step, stabilising the corresponding mRNA is an alternative strategy to increase enzyme abundance, and consequently metabolite levels. In *Oryza sativa*, a tetratricopeptide repeats (TPRs)-containing protein has been identified as a regulator of mRNA stability for various *PAL* genes. Consistent with this role, elevated *PAL* mRNA levels were observed in the corresponding mutant [52]. However, the study did not measure the resulting PAL activity or levels of phenylpropanoid-derived compounds.

A different approach to improve the rate-limiting step focuses on preventing the degradation of the rate-limiting enzyme rather than increasing its production. As is the case for many proteins, the turnover of PAL is regulated by the ubiquitin/26S proteasome degradation pathway, and modulating this pathway can increase PAL levels. In Arabidopsis, KELCH REPEAT F-BOX (KFB) proteins are involved in PAL ubiquitination, which is the initial step towards its degradation by the 26S proteasome [36]. While single *kfb* mutants (i.e. *kfb1*, *kfb20,* and *kfb50*) exhibited no significant metabolic difference compared to wild-type plants, triple *kfb* mutants showed a 2.5-fold increase in PAL activity, resulting in elevated levels of sinapoyl esters and anthocyanins [53]. However, similar to the *PAL2* overexpression in tobacco cells mentioned above, no difference in flavonoid content was observed.

### Minimising the loss of pathway intermediates/end products

In many metabolic pathways, intermediates function as branching points, redirecting carbon skeletons into diverse subsidiary pathways. Within the phenylpropanoid pathway, this branching underpins the biosynthesis of a broad spectrum of phenylpropanoid-derived compounds [35]. However, these diversion points also result in substantial losses of carbon skeleton, thereby reducing the availability of these resources for the synthesis of desired target phenylpropanoids. Minimising these losses can substantially increase the yield of the desired end product. A notable example of this strategy can be found in the domain of decorative rose breeding. Roses lack violet to blue flower varieties due to the absence of the *FLAVONOID-3′5’-HYDROXYLASE* (*F3′5’H*) gene, which is essential for the synthesis of delphinidin-based blue pigments [54]. By downregulating the endogenous *DIHYDROFLAVONOL REDUCTASE*, a gene involved in the synthesis of red and yellow pigment flavonoids, and simultaneously overexpressing the *Viola F3′5’H* gene, roses exhibiting a blue hue were created due to the exclusive accumulation of delphinidin in the petals [54].

Inactivating undesired branching pathways has become more straightforward with the implementation of the CRISPR/Cas9 gene-editing toolkit. To enhance soybean resistance to the soybean mosaic virus, the CRISPR/Cas9 system was employed to simultaneously target three flavonoid biosynthesis genes: *ISOFLAVONE SYNTHASE*, *FLAVANONE-3-HYDROXYLASE*, and *FLAVONE SYNTHASE II* [55]. This strategy redirected the carbon skeletons towards the isoflavones and away from the flavones, flavonols, or anthocyanins. Consequently, the engineered soybean plants exhibited a doubling of the concentration of isoflavones in their leaves compared to wild-type plants, effectively enhancing protection against viral infections [55].

In some instances, the objective of inactivating side branches is not solely to redirect the carbon flux towards desired target compounds but to eliminate the production of specific undesirable metabolites. A notable application of this strategy is the cultivation of non-pungent peppers (*Capsicum sp.*). The characteristic spiciness of peppers is primarily attributed to capsaicin, a compound synthesised from precursors branching from the phenylpropanoid pathway at the level of feruloyl-CoA. By strategically disrupting this metabolic branch, researchers successfully suppressed capsaicin synthesis, resulting in peppers that retained their other qualities while exhibiting a non-pungent phenotype [41].

Another strategy to avoid the loss of pathway intermediates to branching pathways involves altering the plant’s conjugation mechanisms of specific target compounds. For instance, glycosylation is a common modification for phenylpropanoids and involves the attachment of sugar moieties to the core structure [56]. This process not only alters the solubility of these compounds, but can also significantly influence their biological activity and transport within the plant [57]. By inhibiting glycosylation, the concentration of the desired metabolites can be increased. This was demonstrated in soybean, where a metabolic engineering approach was utilised to enhance plant resistance to leaf-chewing insects such as *Helicoverpa armigera* and *Spodoptera litura.* In this instance, the inactivation of *GmUDP-GLYCOSYLTRANSFERASE* (*UGT*) led to the accumulation of the isoflavonoids eupatilin, ononin, isoformononetin, and daidzein [58], subsequently enhancing the plant’s insect resistance.

### Minimising the negative impact of bioactive phenylpropanoids on plant growth

Many specialised metabolites exhibit bioactivity, and alterations in their abundance can profoundly influence plant growth [34]. While modifications in the underlying biosynthetic pathway often drive changes in metabolite levels, the intricate interconnectivity of metabolic networks can also induce unforeseen shifts, potentially leading to the accumulation of toxic by-products [59]. Finally, introducing non-native genes or ectopic gene expression may result in the synthesis of novel compounds or the dysregulation of endogenous metabolic pathways, further complicating metabolic engineering efforts.

For phenylpropanoids, the bioactivity of many metabolites is well-documented, and modifying the pathway can have direct consequences on plant development [59]. For example, the lignan pinoresinol, a dimeric coupling product of coniferyl alcohol, plays a role in the phenotype of Arabidopsis low-lignin mutants overexpressing *F5H*. These mutants exhibit severe defects in lateral root development, which can be mitigated by supplementing the plants with this lignan, suggesting the requirement of pinoresinol or a downstream metabolite for normal root development [60]. Cinnamic acid on the other hand is an example of a phenylpropanoid causing growth defects upon accumulation. It exists in both *cis-* and *trans*-isomeric forms in nature, but only the *trans*-isomer is channelled in the phenylpropanoid pathway [15]. When the pathway is obstructed at the level of C4H, *trans*-cinnamic acid accumulates [61]. Under UV-containing light, *trans*-cinnamic acid is partly converted into its *cis*-form, which acts as a potent auxin efflux inhibitor [15]. This phenomenon may underlie some of the developmental defects observed in *c4h* mutants [61, 62]. Other phenylpropanoid-derived metabolites, such as quercetin and coumarin, are also known to affect auxin transport, and their accumulation might similarly affect plant growth [63, 64].

To prevent the toxic accumulation of bioactive metabolites, plants employ detoxification mechanisms. In phenylpropanoid metabolism, conjugation with sugars or amino acids is a common strategy, as seen in *c4h* mutants, where cinnamoyl-malate and cinnamoyl-hexose accumulate as detoxification products of cinnamic acid [61]. The importance of phenylpropanoid detoxification by glycosylation is further illustrated by the silencing of *UGT88F1*, a phloretin-specific glycosyltransferase in apple (*Malus domestica*). This mutant has severe growth defects that were linked to an overall repressed phenylpropanoid pathway and a disturbed auxin patterning. Feeding the mutant with the glycosylated phloretin phloridzin partly restored the wild-type phenotype [65].

Interestingly, besides acting as a detoxification mechanism, UGTs can also indirectly enhance the yield of target metabolites. For example, overexpression of *UGT79B2 and UGT79B3*, encoding cyanidin glycosyltransferases, resulted in Arabidopsis plants with a deeper purple colouration due to increased anthocyanin levels, whereas *ugt79B2/3* double mutants showed reduced anthocyanin levels [66]. Although these results initially appear counterintuitive when considered solely in the context of enzymatic activity, they can be explained by a feedback activation mechanism regulating the expression of biosynthetic genes. Such interplay between enzyme activity and gene expression can lead to unexpected metabolic fluxes and compound accumulation patterns, introducing significant challenges in predicting the outcome of metabolic engineering strategies.

In addition to conjugation, subcellular compartmentalisation is a critical mechanism for mitigating toxicity. In phenylpropanoid metabolism, sequestration into vacuoles serves as a key mechanism to prevent the excessive accumulation of potentially harmful intermediates in the cytosol [67]. Alternatively, certain phenylpropanoids are actively released into the rhizosphere to avoid reaching cytotoxic concentrations. Within the rhizosphere, these molecules contribute to essential physiological processes, including nutrient acquisition, allelopathy, and plant–microbe interactions [68, 69]. In the case of coumarins such as scopoletin and fraxetin, the ATP-BINDING CASSETTE (ABC) transporter ABCG37 has been identified as a key mediator of their secretion [70]. From a metabolic engineering perspective, identifying and characterising these transporters is important, as they can facilitate the extrusion of toxic metabolites from the cell. This process not only prevents harmful intracellular accumulation, but also minimises the risk of generating undesired by-products and potentially alleviates feedback inhibition, thereby enhancing metabolic efficiency. This concept was exemplified in transgenic Arabidopsis plants overexpressing *ChPLT3*, encoding a caffeyl alcohol transporter identified in *Cleome hassleriana*. Compared to control plants, these transgenic lines exhibited an increased resistance to toxic levels of caffeyl alcohol [71]. Finally, the extrusion of metabolites from the cell can also serve as a strategic approach to streamline downstream processing, particularly in biotechnological applications involving plant cell cultures. By facilitating the controlled release of target compounds into the extracellular environment, this mechanism reduces the need for complex extraction procedures and enhances the overall process efficiency [72].

Despite their importance, many conjugation enzymes and transporters involved in phenylpropanoid metabolism remain unidentified. This knowledge gap currently limits their application in metabolic engineering strategies aimed at optimising the production of valuable phenylpropanoid-derived compounds.

### Organising the pathway enzymes into metabolons

To enhance the rate of carbon flux through metabolic pathways, enzymes can physically associate to form metabolon complexes [73]. Such arrangements facilitate the direct channelling of intermediates between enzyme active sites, minimising their diffusion in the cellular environment. By increasing the turnover efficiency of metabolic conversions, metabolons can prevent the potentially hazardous accumulation of toxic or bioactive pathway intermediates. Consequently, promoting metabolon formation through enhanced enzyme interactions can boost the production of metabolites.

Numerous studies have demonstrated that a subset of phenylpropanoid pathway enzymes can physically interact to form metabolons at the cytoplasmic side of the endoplasmic reticulum [73]. For instance, C4H functions within a metabolon alongside C3’H, 4CL, and CCR to channel *p*-coumaric acid, produced by C4H, via *p*-coumaroyl-CoA in the phenylpropanoid pathway towards the monolignols (Fig. 1) [74]. Notably, in combination with CHALCONE SYNTHASE (CHS), C4H forms a different metabolon, redirecting *p*-coumaric acid towards flavonoids instead of monolignols, illustrating that carbon skeletons can be directed towards various branching pathways depending on the specific metabolon complex formed [74]. The cytochrome P450 enzymes of the phenylpropanoid pathway (namely C4H, C3’H, and F5H) do not interact directly. Instead, they are brought into close proximity by membrane steroid-binding proteins (i.e. MSBP1), which serve as scaffolds or structural backbones of the metabolon [75]. This arrangement provides a platform that facilitates efficient enzyme interaction and cooperation.

One of the first successful applications of scaffold proteins channelling the carbon flux between successive enzymes was to increase the production of the terpenoid mevalonate. By linking the mevalonate biosynthetic enzymes to scaffolds, utilising high-affinity mammalian protein–protein interaction domains, a 77-fold increase in mevalonate production was obtained [76, 77]. Despite its demonstrated success, this approach has yet to be applied to the enhancement of phenylpropanoid production. This is largely due to the inherent challenges of constructing active metabolon complexes. The approach demands precise control over protein folding, stability, and assembly to ensure the functional integrity of the enzymes, as well as the optimisation of substrate channelling mechanisms to achieve an efficient metabolic flux [78, 79]. Overcoming these obstacles necessitates not only a deep understanding of the structure, function, and regulation of the native enzymes involved, but also the careful design and engineering of synthetic complexes.

A simpler strategy to bring successive enzymes into close physical proximity involves the use of translational fusions to combine two subsequent enzymes of a pathway. As for metabolon complexes, translational fusions enable the precise spatial organisation of catalytic activities, which can lead to enhanced reaction rates and a more robust metabolic performance in engineered systems. Such a new-to-nature protein was generated by fusing *CHALCONE ISOMERASE* (*CHI*) and *ISOFLAVONE SYNTHASE* (*IFS*). Implementation of the resulting chimeric enzyme in *Saccharomyces cerevisiae* and *tobacco* resulted in the production of the isoflavonoids daidzein and genistein [80]. However, no comparison was made between the performance of the fused CHI-IFS and their non-fused counterparts.

### Modulating the carbon flux through transcription factors

Gene expression is a highly regulated process in which transcription factors play a central role. By binding to specific DNA motifs within the promoter region of target genes, they can function as either activators or repressors of gene expression. Activators enhance transcription by facilitating the recruitment of RNA polymerase and other components of the transcriptional machinery, while repressors inhibit transcription by obstructing the assembly of the transcriptional complex [81]. The interplay between activators and repressors precisely regulates the expression of genes involved in various pathways. The capacity of transcription factors to concurrently modulate the activity of multiple genes underscores their utility in metabolic engineering.

In the case of the phenylpropanoids, key regulators are members of the *V-MYB MYEOBLASTOSIS VIRAL ONCOGNE HOMOLOG* (*MYB*) and the *NAM, ATAF*, and *CUC* (*NAC*) gene families [82]. The overexpression of these transcription factors presents a promising metabolic engineering strategy to enhance phenylpropanoid production. For instance, the constitutive expression of the native *NAC17* in *V. vinifera* cell suspensions resulted in a 2.5-, 3.5- and 2-fold increase in the accumulation of cyanidin-3-glucose, proanthocyanins and flavonoids, respectively [83]. This metabolic alteration induced a colour shift in the transgenic cells, changing from dark pink to deep purple. Similarly, the ectopic expression of the Arabidopsis *MYB12* transcription factor in tobacco plants resulted in a 40-fold increase in rutin, a threefold increase in kaempferol, and a fivefold increase in quercetin compared with wild-type plants [84].

These examples underscore the significant role of transcription factors in metabolic engineering. However, constitutive expression across the entire plant, leading to metabolite accumulation in all tissues, may not represent an optimal allocation of energy or carbon resources. Thus, inducible or tissue-specific expression systems are often favoured over constitutive expression. The fruit is a frequently targeted organ for enhancing phenylpropanoid content, as many phenylpropanoids possess antioxidant properties. Increasing their concentration in fruits represents a promising strategy for developing nutritionally enriched and health-promoting crops. For example, the expression of *MYB12* under the control of the fruit-specific promoter in tomatoes led to fruit containing flavonoid and hydroxycinnamate that constituted 10% of the dry weight [85]. This genetic modification not only increased the nutraceutical value of the tomatoes, but also altered their colour from brown to a vibrant blue/purple.

In addition to the use of activators, transcriptional repressors are also strategically employed in metabolic engineering to fine-tune gene expression and enhance the production of target metabolites [86]. In this approach, transcription factors are either partially or fully inactivated, thereby alleviating the repression exerted on the biosynthetic pathway of interest. A notable example of this strategy is observed in *Salvia miltiorrhiza*, where a CRISPR/Cas9-based gene-editing approach was utilised to knock out *bZIP2*. This inactivation resulted in a 1.67-fold increase in both rosmarinic acid and salvianolic acid B levels, demonstrating the effectiveness of transcriptional repressor modulation in improving secondary metabolite yield [87]. Similarly, in Arabidopsis, the mutation of *MYB7*, which encodes a repressor of flavonoid biosynthesis, led to a twofold increase in flavonoid production [88].

### Exploiting elicitors to boost production

Elicitors are defined as molecules, conditions, or organisms that trigger a response in the organism to which they are applied. This response typically manifests as a stress reaction, characterised by the activation of a defence mechanism against potential threats. A key aspect of this defence response is the enhanced production of specialised metabolites, which has generated significant interest in utilising elicitors to augment the production of these compounds. Unlike strategies that focus on producing a specific end product, elicitors trigger a broad activation of plant specialised metabolism. This widespread response can sometimes result in less efficient energy and carbon allocation compared to more targeted approaches. However, this broader activation can be beneficial in contexts where multiple metabolic pathways or particular types of specialised metabolites are of interest. Finally, elicitors are not restricted to particular species, offering notable flexibility and a distinct advantage over genetic engineering approaches.

An illustrative example of a chemical elicitor is piperonylic acid (PA). Treatment of plants with PA induces a stress response that confers partial protection against plant-parasitic root-knot nematodes [89]. This response is associated with the reprogramming of phenylpropanoid metabolism, resulting in an increased production of various phenylpropanoid-derived compounds. The shift in metabolism is directly attributable to PA’s capacity to act as an inhibitor of C4H [90], one of the key enzymes of the phenylpropanoid pathway (Fig. 1). Although the positive effect on downstream compounds upon blocking the phenylpropanoid pathway is paradoxical, the effect is likely due to a positive feedback mechanism activated by the transient inhibition of the pathway. In addition, stress hormones, such as jasmonate and salicylic acid, operate through distinct mechanisms, and trigger a cascade of transcriptional regulators, leading to the upregulation of genes encoding enzymes involved in phenylpropanoid biosynthesis. Numerous studies have highlighted the critical role of these hormones in modulating phenylpropanoid production. For example, treatment with methyl jasmonate of field-grown *Rubus ideaus* resulted in a 1.25-fold increase in anthocyanin production and a 1.3-fold increase in total phenolic content [91]. Likewise, treatment with coronatine, a methyl jasmonate analogue, enhanced the levels of caffeic acid, isoferulic acid, *p*-coumaric acid, and sinapic acid in *Lemna paucicostata* [92].

Besides compounds, environmental factors such as temperature, salinity, heavy metals, and UV radiation can induce stress responses in plants, leading to the accumulation of specialised metabolites, including phenylpropanoids. For example, exposure of *Brassica oleracea* to ecologically relevant UV-B radiation resulted in significant increases in the levels of kaempferol and quercetin [93]. Similarly, the supplementation of metal ions to the growth medium has been shown to stimulate phenylpropanoid biosynthesis. In a study by Cai, Kastell [94], varying concentrations of Co^2+^, Cd^2+^, and Ag^+^ (5, 25, and 50 μM) were used to enhance resveratrol production in *V. vinifera* cell suspension cultures, resulting in a 1.6-fold increase in the yield of 3-*O*-glucosyl-resveratrol across all treatments [94].

In addition to the aforementioned abiotic elicitors, biotic elicitors also play a significant role in modulating plant responses, particularly by influencing secondary metabolism. Microorganisms, including fungi, bacteria, and viruses, can trigger complex signalling cascades in plants, which in turn activate defence mechanisms and enhance the production of specialised metabolites. For example, inoculation of *Albizia kalkora* adventitious root cultures with *Rhizobium rhizogenes* resulted in a 1.8-fold increase in the production of the isoflavonoid genistein after just 6 days of cultivation [95].

The initial triggers of the biotic response are specific compounds such as polysaccharides derived from fungal cell walls, bacterial peptidoglycans, and arthropod exoskeletons. The recognition of these pathogen-associated molecular patterns (PAMPs) by plant receptors triggers a cascade of downstream events, including the activation of key enzymes involved in specialised metabolite biosynthesis. This highlights the potential use of PAMPs, rather than the entire organism, to stimulate the production of target metabolites. For instance, elicitation of *Hypericum perforatum* cell cultures with biotic agents such as chitin, pectin, or dextran resulted in the elevated production of phenolics, flavonoids, flavanols, and anthocyanins [96]. Among these, pectin was particularly effective, doubling the flavonoid content within seven days. Similarly, treatment of *H. perforatum* cell suspension cultures with a fungal cell wall extract from *Colletotrichum gloeosporioides* led to a remarkable sixfold increase in xanthone content [97]. Notably, xanthone accumulation was further enhanced when the cultures were treated with methyl jasmonate prior to the addition of the fungal extract. This demonstrates that combining multiple elicitors can synergistically amplify the production of specialised metabolites, providing a robust strategy for metabolic engineering and secondary metabolite production.

### Introduce additional pathway entry points

Instead of increasing the supply of carbon along the canonical pathway, an alternative entry point into the metabolic pathway can be utilised to boost the production of the desired compound.

In the case of the phenylpropanoid pathway, the bifunctional PTAL enzymes offer an alternative entry point in monocots, utilising tyrosine alongside phenylalanine as a precursor for phenylpropanoids. In grasses, up to half of the lignin content is derived from tyrosine, indicating that a significant portion of the pathway’s flux is facilitated through PTAL activity [98, 99]. However, the impact of overexpressing grass *PTAL*s on phenylpropanoid metabolism in dicots, which naturally lack this enzyme, remains unexplored.

Certain bacteria possess monofunctional TALs, which, unlike PTALs, exclusively utilise tyrosine as their substrate [100]. Employing these enzymes in plants may mitigate metabolic competition for phenylalanine. In addition, TALs also bypass the production of cinnamic acid, making it unlikely they will be affected by the feedback mechanisms developed in plants based on the substrate and product of PAL (i.e. phenylalanine and cinnamic acid, respectively, Fig. 1). The efficacy of TALs in engineering the phenylpropanoid pathway has been demonstrated by the ectopic expression of a *TAL* from *Rhodobacter sphaeroides* in Arabidopsis, leading to the accumulation of anthocyanins in 5-day-old seedlings and quercetin glycosides, sinapoyl, and *p*-coumaroyl derivatives in 21-day-old seedlings [101]. Over the years, an increasing number of bacterial TAL enzymes have been characterised [100], and it might be valuable to investigate whether some of these enzymes could further enhance the production of desired phenylpropanoids.

Apart from the PAL- and TAL-based entry points, emerging evidence suggests the existence of alternative metabolic routes that may expand the potential for engineering this pathway. Notably, isotopic labelling studies conducted in *Camellia sinensis* have revealed a previously unrecognised PAL-bypass pathway [102]. In this route, cinnamate is synthesised from phenylalanine through intermediates such as phenylpyruvic acid and phenyllactic acid, bypassing the direct deamination of phenylalanine by PAL. Despite compelling biochemical evidence, the genes encoding the enzymes responsible for this bypass pathway remain uncharacterised.

### Increase the supply of precursors

One of the key determinants influencing the final yield of the desired metabolite(s) is the availability of precursors. Given that the phenylpropanoid pathway starts from primary metabolism, this strategy underscores the importance of understanding the interdependence between primary and specialised metabolic pathways to effectively maximise product yield.

The precursors of the phenylpropanoid pathway are supplied by the shikimate pathway, which converts phosphoenolpyruvate and erythrose 4-phosphate to chorismate in seven enzymatic steps. Subsequently, chorismate is further metabolised via the intermediate arogenate, ultimately leading to the formation of phenylalanine and tyrosine [103]. Increasing the production of phenylpropanoid precursors can enhance the carbon flux towards the phenylpropanoid pathway. This objective can be achieved by employing strategies targeting the shikimate pathway, analogous to those described for the phenylpropanoid pathway.

For instance, the allosteric feedback inhibition of AROGENATE DEHYDRATASE (ADT) represents a critical regulatory bottleneck that typically restricts the carbon flux through the post-chorismate branch of the shikimate pathway. In *Petunia hybrida* flowers, high phenylalanine concentrations, essential for sustaining elevated levels of phenylpropanoid volatiles, are achieved through a feedback-relaxed ADT enzyme, circumventing this limitation [104]. Overexpression of a feedback-relaxed ADT in Arabidopsis led to a significant accumulation of phenylalanine, accompanied by a twofold increase in anthocyanin levels [105].

In grasses, a comparable adaptation exists for tyrosine biosynthesis: feedback-insensitive forms of AROGENATE DEHYDROGENASE (ADH) have evolved to support the dual phenylalanine/tyrosine-derived phenylpropanoid pathway [106]. Transient overexpression of these ADH variants from *Sorghum* and *Brachypodium* in tobacco resulted in increased tyrosine accumulation. However, since this bypass is relevant only in species possessing the tyrosine-derived branch of phenylpropanoid biosynthesis, its application is limited to such systems. To date, the impact of overexpressing these ADH genes directly in grasses has not yet been evaluated.

Additionally, feedback-insensitive enzymes from bacteria have also been employed for the metabolic engineering of the phenylpropanoid pathway. For example, the overexpression of a feedback-insensitive *3-DEOXY-D-ARABINO-HEPTULOSONATE 7-PHOSPHATE SYNTHASE* (*DAHPS*) gene, which encodes the first committed enzyme of the shikimate pathway, resulted in increased levels of phenylalanine, tyrosine, and various phenylpropanoids in both Arabidopsis and *Nicotiana tabacum* [107, 108]. Additionally, several feedback-insensitive forms of the Arabidopsis DAHPS were recently identified via through a forward genetic screen using ethyl methanesulfonate (EMS) mutagenesis [109]. Although these variants led to the accumulation of phenylalanine and tyrosine, phenylpropanoid levels in the corresponding plants remained unchanged.

Enhancing the availability of precursors can also be achieved by leveraging species-specific adaptations at the interface between primary and specialised metabolism. A notable example is provided by AROMATIC AMINO ACID HYDROXYLASES (AAHs) identified in the moss *Physcomitrium patens* and the gymnosperm *Pinus taeda* [110]. These enzymes catalyse the hydroxylation of phenylalanine to tyrosine, thereby playing a pivotal role in modulating the balance of precursor availability for the downstream phenylpropanoid pathway. In *P. patens*, loss of AAH activity resulted in the accumulation of phenylalanine and caffeic acid esters [110]. These findings highlight how species-specific metabolic interfaces can be targeted to optimise precursor allocation, offering opportunities to enhance phenylpropanoid production.

In addition to strategies aimed at increasing the precursor pool, exploiting subcellular pathway compartmentalisation offers a promising approach to enhance metabolic flux towards target pathways. In the case of the phenylpropanoid pathway, a distinct spatial separation exists between the site of precursor synthesis and downstream metabolic processes. Enzymes involved in phenylpropanoid biosynthesis are localised in the cytosol, while the aromatic amino acid precursors required for this pathway are synthesised primarily within the plastids. Consequently, aromatic amino acids must be transported from the plastids to the cytosol to support the phenylpropanoid pathway. This translocation is facilitated by transmembrane amino acid transporters, such as CATIONIC AMINO-ACID TRANSPORTER (CAT) proteins, which can be exploited to enhance the carbon flux towards the phenylpropanoid pathway. The overexpression of a native *CAT* in *P. hybrida* resulted in an increased production of phenylalanine-derived volatiles, including benzaldehyde, eugenol, vanillin, and phenylethylbenzoate [111].

Besides the genetic strategies to increase the precursor availability, the limiting precursor can also be directly provided to plants. The simplicity and relatively low upfront cost make such feeding approach a popular strategy to increase the concentration of downstream metabolites. For instance, the addition of 1 mM of phenylalanine to a cell suspension culture of the medicinal plant *Rhodiola imbricata* resulted in a 2.5-fold increase in the production of rosavins, a group of cinnamyl mono- and diglycosides [112]. However, higher concentrations of phenylalanine negatively affected the yield, highlighting the necessity of optimising the precursor concentration to mitigate potential toxicity effects.

The application of precursor feeding can also be extended to whole plants. For instance, chrysanthemums and anemones, which generally lack a prominent floral fragrance, exhibited a distinguishable enhancement in scent upon treatment of cut flowers with phenylalanine, due to an increase in benzoic phenylpropanoids [113]. Similarly, soaking entire *P. hybrida* plants in a phenylalanine-containing solution resulted in the accumulation of caffeate, ferulate, rosmarinate, and phenylethylamine [114]. Comparable outcomes were achieved through foliar application of phenylalanine, indicating that spraying could offer a practical alternative for large-scale agricultural operations, given its convenience for field implementations.

## Future perspectives and transdisciplinary innovations

In recent decades, plant metabolic engineering strategies are addressing diverse needs and objectives. For example, farmers are particularly interested in engineered plants with increased resistance to biotic and abiotic stresses, or reporter lines that can be used to monitor such stress conditions [14, 45]. Consumers, on the other hand, are interested in plants with enhanced nutraceutical value, such as foods with an increased antioxidant content, or altered phenotypical traits such as flower colour and fragrance [115–117]. For scientific or industrial applications, pathway engineering is employed to modify plants towards versatile toolkits. An interesting example is the use of plants as bioindicators for land mines [118]. In this case, plants are engineered to accumulate anthocyanins upon detecting the presence of explosives in the soil, changing their leaves from green to purple.

Among the metabolic engineering strategies we reviewed, precursor feeding, elicitor treatment and the strategic manipulation of transcription factors have emerged as particularly effective in enhancing phenylpropanoid production. However, the other strategies, while significantly underexplored in the context of the phenylpropanoid pathway, should not be dismissed for the reason of lacking potential. Advances in our understanding of plant specialised metabolism, along with the identification of novel enzymes with interesting features (i.e. lack of feedback inhibition), could generate innovative metabolic engineering approaches.

In addition to individual strategies, the simultaneous application of multiple strategies has the potential to achieve better results. However, determining the optimal configuration for combining different methodologies presents significant challenges. The complexity of metabolic networks, regulatory intricacies, and metabolic burden all contribute to the formidable task of metabolic optimisation [20, 26]. Addressing these challenges requires an iterative, multidisciplinary approach that integrates genetics, biochemistry, and engineering principles. In specific instances, high-throughput screening can be utilised to identify optimal conditions that maximise the yield of target metabolites [119]. For example, when combining precursor feeding with elicitor treatments, plant cell suspension cultures can be easily employed in a high-throughput screening platform to efficiently determine the optimal compound combinations [120]. These platforms, leveraging automated processes and advanced analytical techniques, not only expedite the experimental workflow but also enhance the reliability and reproducibility of results [121]. However, the high costs associated with maintaining cell cultures, the variability in yields influenced by specific cell lines and growth conditions, and the challenges of ensuring stability in transgenic lines when employing genetic strategies remain significant obstacles.

While we have emphasised the advantages of using plants as hosts for producing various phenylpropanoids, it is crucial to recognise the benefits microorganisms have to offer. Given their small size, rapid growth, and high transformation efficiency, microorganisms are particularly valuable for large-scale screening for phenylpropanoid pathway engineering [122]. For instance, a recent high-throughput screen in *E. coli* of a library of mutated versions of *Anabaena variabilis* PALs identified a feedback-insensitive PAL variant with a sixfold reduction in cinnamic acid affinity [123]. Microorganisms have also been instrumental in providing mechanistic understanding of enzymatic conversions. Research in microorganisms revealed that the substrate specificity of ammonia lyases is determined by a single amino acid within the catalytic pocket, referred to as the substrate specificity site [124]. For PALs, this amino acid is phenylalanine, while in TALs it is histidine. By replacing phenylalanine with histidine in Arabidopsis PAL1, the substrate specificity of heterologous-produced enzymes was shifted from phenylalanine to tyrosine [124]. Recent advancements in precision engineering using CRISPR/Cas9 now allow similar amino acid substitutions directly in plants, thereby effectively converting a native PAL enzyme into a (P)TAL. This approach offers the advantage of bypassing the need to introduce foreign genes into the genome, thereby mitigating potential complications such as position effects associated with inserted DNA. Furthermore, this gene-editing strategy enables highly specific and minimal modifications, distinguishing it fundamentally from traditional transgenic methods. These key differences have significant practical implications, particularly in terms of regulatory approval, stability, and the precision of genetic alterations.

In addition to providing fundamental insights into enzymes and metabolic pathways, microorganisms have also played a key role in the development of metabolic engineering tools. Notably, multiple biosensors serving as proxies for the concentration of specific metabolites have been developed in microorganisms to facilitate easy readouts during enzyme optimisation processes. For example, a biosensor derived from a *Pseudomonas putida* transcription factor was engineered in *E. coli* to detect the stilbene resveratrol, which enabled the efficient screening of a mutagenised Arabidopsis *4CL* library to enhance resveratrol production [125]. Over the past decade, various other phenylpropanoid biosensor systems have been created, targeting flavonoids such as naringenin [126], kaempferol [127], and quercetin [128]. In this context, the substantial advancements achieved in microorganisms have established a foundation for the development and application of analogous tools in plant systems. For example, a self-sustained bioluminescence reporter system was successfully engineered in tobacco by integrating a fungal bioluminescence gene cluster. This system leverages the endogenous presence of caffeic acid as a substrate to synthesise luciferin, thereby enabling a bioluminescent output [129, 130]. This innovation allows the non-invasive monitoring of metabolic processes. When applied to crops, such bioluminescent plants would enable farmers to monitor plant health and detect pest presence more effectively, facilitating timely interventions and hence reducing crop losses.

Although findings from studies in microorganisms are frequently translated into plants, research in plant systems also offers significant opportunities to advance metabolic engineering in microorganisms. This is especially pertinent in understanding how plants effectively manage toxic metabolic intermediates and end products using glycosylation and compartmentalisation [122]. These sophisticated strategies employed by plants provide valuable insights that can be leveraged to enhance microbial metabolic engineering systems and optimise the production yield of target compounds. For instance, the overexpression of plant-derived enzymes such as glycosyltransferases in microbial hosts has been shown to modify the physicochemical properties of specialised metabolites, improving their solubility and bioactivity, and reducing their toxicity [56]. Furthermore, the incorporation of plant-specific metabolite transporters into microbial systems could mitigate the intracellular accumulation of toxic intermediates or end products by facilitating their sequestration or export, thus enhancing overall metabolic performance. Another promising avenue involves the engineering of metabolon-like enzyme complexes inspired by plants, which could channel intermediates more efficiently, reduce metabolic bottlenecks, and limit the toxicity associated with intermediate build-up. Similarly, adopting subcellular compartmentalisation that mimics organelle-specific isolation of metabolic processes can enhance the yield of specialised metabolite production by improving pathway flux and minimising metabolic crosstalk. In bacteria, such compartmentalisation has been successfully engineered using synthetic organelle-like systems, while in yeasts, native organelles have been exploited for this purpose [72, 131–133]. These strategies have been effectively applied to the production of various metabolites, however they have not yet been implemented for phenylpropanoids. These cross-kingdom applications of metabolic engineering principles highlight the untapped potential of leveraging plant-derived mechanisms to drive significant advancements in microbial biotechnology, fostering innovations in the sustainable production of high-value metabolites.

Finally, the cocultivation of plants and microorganisms could combine the advantages of both organisms to increase the yield of target compounds. This approach not only enables the exploitation of the optimal attributes of each species, but also mitigates the metabolic burden on individual organisms, reduces interference among different pathway branches, and potentially bypasses feedback inhibition. A notable example is the laboratory strain PCC 6803 of the cyanobacterium *Synechocystis* sp., which has been successfully adapted to produce and secrete significant quantities of phenylalanine into its extracellular environment [134]. When co-cultivated with plant cells, such engineered *Synechocystis* strains could serve as an efficient source of phenylalanine, thereby enhancing the biosynthesis of phenylalanine-derived specialised metabolites in plant systems. This approach represents a promising avenue for synergistic metabolic engineering, leveraging plant–microbe interactions to achieve higher yields of target compounds.

## Conclusion

Plants offer a unique and versatile platform for the production of specialised metabolites. They possess the enzymatic machinery and metabolic pathways necessary to synthesise complex molecules that are often challenging to produce via chemical synthesis or microbial fermentation. Additionally, plants can generate a wide variety of structurally diverse metabolites, reflecting the evolutionary optimisation of these pathways to address ecological interactions and environmental challenges. Advances in plant biotechnology and metabolic engineering have further enhanced their utility, enabling the targeted overproduction of specific compounds and the introduction of novel biosynthetic pathways. Leveraging the vast metabolic diversity across the tree of life alongside novel biotechnological innovations allow the development of powerful tools to drive future innovations. Finally, the convergence of metabolic engineering approaches in both plants and microorganisms presents unprecedented opportunities for synergy. Together, these innovations contribute to the growing potential of plants as sustainable and efficient biofactories, providing an environmentally friendly platform for producing a diverse array of high-value specialised metabolites with applications in medicine, agriculture, and industry.

## Data Availability

No datasets were generated or analysed during the current study.
